# Comment on: ‘Reduced Temporal Muscle Thickness Predicts Shorter Survival in Patients Undergoing Chronic Subdural Haematoma Drainage’ by Korhonen et al.

**DOI:** 10.1002/jcsm.13621

**Published:** 2024-10-25

**Authors:** Xiaolin Du, Guangtang Chen, Zeguang Ren

**Affiliations:** ^1^ Department of Neurosurgery The Affiliated Hospital of Guizhou Medical University Guiyang Guizhou Province China; ^2^ Department of Neurosurgery The Jinyang Hospital Affiliated to Guizhou Medical University Guiyang Guizhou Province China

We have taken a keen interest in the recent article titled ‘Reduced temporal muscle thickness predicts shorter survival in patients undergoing chronic subdural haematoma drainage’ by Korhonen and colleagues [1]. The study reveals a significant correlation between the reduced temporal muscle thickness (TMT), measured preoperatively via computed tomography (CT) scans and the shortened postoperative survival period in patients who have undergone chronic subdural haematoma (CSDH) drainage surgery. The authors have conducted a comprehensive study, incorporating a range of considerations such as age, gender, BMI, imaging indicators and surgical methods, leading to convincing conclusions. However, we have noticed that several important factors may have been overlooked in the execution of the study. We would like to offer the following suggestions for the authors' consideration.

Firstly, TMT, serving as an emerging surrogate marker for muscle mass, function and nutritional status, can be measured through CT, magnetic resonance imaging (MRI) and ultrasound examinations. While the authors have nearly perfected the consideration of TMT measurement, we believe that using two or more imaging methods simultaneously could yield even better results. In addition to TMT, the literature has reported the temporal muscle area (TMA) and temporal muscle volume (TMV) as new surrogate markers related to the temporal muscle and skeletal muscle mass [2]. If the assessment of the temporal muscle in relation to CSDH prognosis were to combine all three—TMT, TMA and TMV—the results would be more compelling. Although a standard method for measuring TMT has not yet been established, nor has an artificial intelligence‐based method been used for TMT measurement, several studies have used volume rendering software to measure TMV [3, 4, 5]. There have also been reports of deep learning‐based quantification methods for TMA [6]. Furthermore, plasma protein levels can reflect the body's protein nutritional status, the severity of disease and the risk of surgery, with common indicators including albumin, prealbumin, transferrin and retinol‐binding protein, especially the latter three being more sensitive and effective indicators of nutritional status [7, 8]. To our knowledge, no studies have reported on the relationship between plasma protein levels and CSDH prognosis, and the authors may wish to explore this further.

Secondly, this is a retrospective study that included only high‐priority variables in the multivariate analysis. We have observed that there are many factors affecting the prognosis of CSDH patients, including age, gender, frailty, malnutrition, cancer, hypertension, diabetes, blood diseases, and the use of anticoagulant or antiplatelet aggregation drugs and so on [9, 10, 11]. In terms of imaging signs, factors affecting the prognosis of CSDH patients are not limited to hematoma volume and midline shift, but also include hematoma thickness, hematoma type, outer membrane formation, residual volume in the hematoma cavity and so on [10, 12]. In addition, postoperative complications such as brain tissue damage, pneumocephalus, acute subdural haemorrhage and epilepsy are also factors affecting the prognosis of CSDH [10, 13]. Therefore, we believe these factors should not be overlooked. In addition, although the authors analysed the body mass index (BMI), we suggest including weight changes in the predictive model to potentially reveal new findings. Also, the authors only counted the number of deaths, and the specific causes of death were not described in detail, which may have an impact on the final results.

Thirdly, we found that the surgical methods for CSDH patients were not completely unified. Most patients underwent cranial drilling, while some patients underwent craniotomy, with room temperature saline rinse during surgery, followed by placement of a subdural drainage tube. Studies have shown that warming the irrigation fluid temperature to body temperature during cranial drilling for CSDH can reduce the risk of recurrence by more than half, so body temperature irrigation should become the standard treatment [14]. And the pipeline can adopt non‐subdural drainage (subaponeurotic or subperiosteal), this method does not only increase the recurrence rate but also can avoid iatrogenic brain tissue damage [15]. This can optimize the prognosis of CSDH patients. In addition, the author's unit tends to use general anaesthesia, and meta‐analysis shows that local anaesthesia has the following advantages: shorter operation time, shorter hospital stay and fewer postoperative complications [16]. The above factors are worth considering by the authors, and we look forward to future research to further improve these aspects.

Finally, we would like to thank the authors for this study again, as these important results will be an important factor for neurosurgeons to consider after surgery in the future. Studies have reported that reduced total protein levels at admission are a new factor for poor prognosis after endoscopic removal of intracerebral haemorrhage [17]. Moreover, a high protein intake following subarachnoid haemorrhage can improve TMV, and patients in the TMV maintenance group show significantly correlated functional recovery at discharge compared to the TMV atrophy group [5]. These studies emphasize the importance of high protein intake. Therefore, dynamically assessing the changes in TMT of CSDH patients may help to carry out more effective nutritional treatment, thereby reducing the mortality rate of CSDH patients. However, further prospective studies are needed to confirm this.

## Ethics Statement

The authors certify that they comply with the ethical guidelines for authorship and publishing in the *Journal of Cachexia, Sarcopenia and Muscle* [18].

## Conflicts of Interest

The authors declare no conflicts of interest.
